# Employees’ Environmental Protection and Charitable Donation and Ethical Leadership: An Empirical Study

**DOI:** 10.3390/ijerph16132282

**Published:** 2019-06-27

**Authors:** Qian Zhang, Yun Liu

**Affiliations:** 1 School of Business Administration, Southwestern University of Finance and Economics, Chengdu 611130, China; 2 School of Business Administration, Shanghai Lixin University of Accounting and Finance, Shanghai 201620, China

**Keywords:** ethical leadership, environmental protection, charitable donation, organizational virtuousness, hierarchical linear modeling, multilevel mediation analysis

## Abstract

The sustainable development of the environment and society depends not only on firms’ social responsibility initiatives, but also on employees’ socially responsible behavior during their daily work life. Hence, it is important to study why and how employees go about the socially responsible behaviors (SRB), such as environmental protection and charitable donations. Although research has been done on the antecedents of employees’ SRB from personal, contextual and leadership perspectives, little is known about the mechanism through which they affect these behaviors. Moreover, compared with the other two perspectives, research from the leadership perspective is relatively scarce. In this paper, we aim to fill these research gaps. Based on 936 respondents from 109 corporations, we empirically test the cross-level direct effect of ethical leadership on employees’ SRB and the cross-level mediating effect of perceived organizational virtuousness. In our empirical analyses, we adopt statistical methodologies such as hierarchical linear modeling and multilevel mediation analysis. Our results show that perceived organizational virtuousness partly mediates the influence of ethical leadership on employees’ environmental protection and charitable donation. In other words, ethical leadership enables employees to form the perception of organizational virtuousness, and therefore employees are more engaged in environmental protection and charitable donations. This research provides important insights for firms and their employees to become more socially and environmentally responsible.

## 1. Introduction

Owing to its important role in the sustainability of environment and societies, corporate social responsibility (CSR), including environmental protection and charitable donations, has become an active research field in the past decades. Three approaches have been used by scholars to explore CSR: macro, micro, and individual approaches. The macro approach of CSR studies treats a firm as a personified independent entity and explores its intentions, initiatives and outcomes to take social responsibility at the organizational level [1]. For example, Wu et al. examined the relationship between green CSR and firm innovation performance [2]. Many scholars, however, argue that more attention should be paid on the micro level [3]. The micro approach of CSR studies focuses on how employees’ perception or experience of CSR affects their attitude and behavior. It is concerned with what attitudes and behavioral changes will occur when employees perceive their employers have fulfilled the social responsibility [4]. For example, Kim et al. explored the underlying process of the association between employee’s perceived CSR and employees’ behavior with a moderated mediation model [5]. The third approach of CSR studies is called the individual approach in this article, focusing on the motivation and determinants of employees’ socially responsible behaviors (E-SRB). This individual approach focuses on employees’ discretionary socially responsible behavior at the workplace rather than their employers’ socially responsible behavior. Moreover, the individual approach differs from the micro approach because the micro approach focuses on how the perceived CSR influence employees’ work attitudes and behavior, while the individual approach explores when, how and why employees’ SRB occurs.

SRB are those discretionary decisions and actions taken by individuals in organizations to enhance societal well-being or to avoid harmful consequences for society [6]. Similarly, Roeck and Farooq defined E-SRB as employees’ discretionary behaviors that intend to enhance the well-being of their organization’s stakeholders, including the natural environment [7]. Examples of employees’ SRB in their day-to-day organizational life include volunteer work, donations, saving energy and water, reducing wastage, adhering to the operating procedures and environmental standards [8]. These discretionary activities can potentially contribute positively to the environment and society. For example, employees can help protect the environment by reducing their own energy consumption or greening the operations of their firms. Employees can also donate their used products like cloths and computers so that these products are less likely to end up in landfills potentially polluting the environment. Inthis research, we focus on employees’ SRB of environmental protection and charitable donation.

Although some similar constructs, such as organizational citizenship behavior (OCB) and pro-social behavior, are also discretionary in nature, these kinds of employee behavior is usually more related to coworkers or the organizations, while E-SRB is beyond the organization and towards the society [7]. In the literature, E-SRB is equivalent to the following concepts [7]: employee engagement in CSR [9], employees’ extra-role CSR performance [10], and OCB targeting external stakeholders (e.g., OCB toward the environment [11]).

More recently, the topic of understanding why and how employees engage in SRB towards environmental improvement and charitable donation has been highlighted in the literature [12]. Through literature review, Gond et al. found that researchers have discussed the determinants of E-SRB mainly from personal, contextual and leadership perspectives [13]. For example, from the personal perspective, employees’ socio-demographic characteristics, personality traits and other personal factors such as employees’ values and belief have been proved to have an impact on E-SRB [13]. From the contextual perspective, Slack et al. [9] found that some organizational context factors, such as poor communication, a perceived weak and low visibility of CSR culture and strategic misalignment of CSR to business and personal objectives, serve to explain the diversity of employee engagement with SRB. Wong et al. designed a conceptual framework that links environmental literacy and factors affecting pro-environmental behavior [14]. Afsar et al. found that perceived CSR has both a direct and an indirect influence, through organizational identification, on pro-environmental behavior [15]. Grant explained how depleted task, social, and knowledge characteristics of jobs trigger compensatory motives during initial volunteering episodes [16].

Although many researchers have explored the antecedents of employees’ SRB, few studies have explored the mechanisms through which they affect this kind of behaviors [17]. In addition, compared with the other two perspectives, the research on the leadership perspective is relatively limited [18]. Therefore, to fill these research gaps identified above, this paper explores the determinants of employees’ SRB and the mechanisms through which they affect these behaviors from the perspective of ethical leadership.

The major contributions of this research are the following: First, previous studies mainly focused on the impact of transformational leadership on employees’ SRB, ignoring the role of ethical leadership. Second, this study deeps our understanding on how leadership style influences employees’ SRB by exploring the mediating effect of perceived organizational virtuousness between the two. Third, many earlier studies discussed how employees’ perception of a certain leadership style affect their SRB and focused on individual-level analysis. In contrast, this article treats ethical leadership as a group variable and explores the cross-level influence of ethical leadership on employees’ SRB, as well as the cross-level mediating effect of perceived organizational virtuousness (POV).

The rest of the article is organized as follows: In Section 2, the theoretical framework and research hypotheses are proposed based on a literature review. Section 3 details the research methodology, followed by the empirical analyses and results. Subsequently, managerial implications are discussed. Finally, main conclusions are drawn and the limitations of this study and suggestions for future research are put forward.

## 2. Theoretical Framework

Although many studies link leader behaviors to firm-level CSR [19], few studies have examined the effect of leadership style on employees’ SRB. However, evidence does show that some leadership variables can impact employee’s SRB. For example, Ramus and Steger showed that supervisory support behavior (as well as corporate environmental policy) influences the probability that employees will try to innovatively solve environmental problems [20]. Vlachos et al. found that managers’ CSR judgments have cascading effects on employee CSR judgments, and managers’ involvement in implementing deliberate strategy can strengthen or weaken these cascading effects [10]. Wesselink et al. concluded that leadership behavior (as exemplary behavior) can affect the employees’ pro-environmental behavior [21].

Inspired by these findings above, some scholars began to explore the influencing factors of employees’ SRB from the perspective of leadership style. Roeck and Farooq examined an integrated moderated mediation model in which employees’ perception of ethical leadership moderates the mediating mechanism between their perceptions of CSR, organizational identification, and employees’ SRB [7]. Groves and Larocca found that transformational leadership is positively related with subordinates’ valuation of CSR [18]. Graves et al indicated that the environmental transformational leadership provided by employees’ managers is associated with employees’ autonomous motivation which is in turn positively related to pro-environmental behaviors [22]. The empirical study of Robertson showed that the environmentally-specific transformational leadership is positively related to subordinates’ workplace pro-environmental behaviors [23]. The research result of Afsar et al. lent support for the interactive effect of environmentally specific servant leadership with CSR in predicting employee pro-environmental behaviors [15]. Robertson and Carleton revealed that environmentally-specific transformational leadership directly affects employees’ voluntary green behaviors, and that this relationship is partially mediated by employees’ perceptions of their coworkers’ pro-environmental climate, and that employees’ environmental locus of control moderates the effect of pro-environmental climate perceptions of coworkers [24]. Robertson and Barling found that environmentally transformational leadership indirectly affects employees’ workplace pro-environmental behaviors by influencing employees’ harmonious environmental passion [25].

From the review above, it can be seen that existing studies about the predictors of employees’ SRB from the leadership perspective mainly focus on transformational leadership. These studies explore the impact of transformational leadership on employees’ SRB and its influencing mechanism. Although several studies mentioned the effect of the other leadership styles (such as servant leadership and ethical leadership) on employees’ SRB, these leadership styles mentioned above are only regarded as moderators in research model. As for the influencing mechanism of leadership style on employees’ SRB, the following mediating variables, such as pro-environmental work climate of coworkers [24], employees’ harmonious passion for the environment [25], and employees’ autonomous and external motivation [22], have been used to explain how environmentally transformational leadership affects employees’ pro-environment behavior.

**Figure 1:**
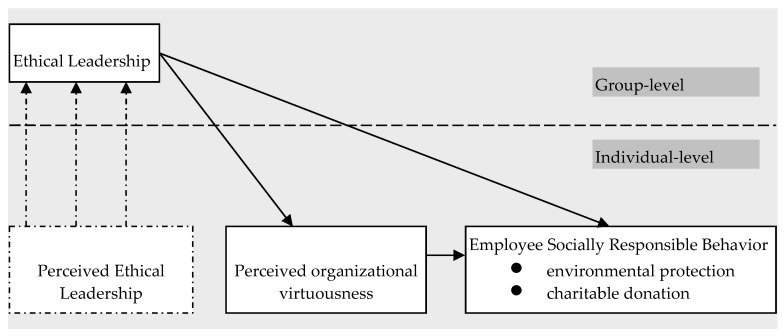
Theoretical Model.

This research intends to explore the determinants of employee’s SRB from the leadership perspective. Because SRB has ethical attributes in essence, so, using transformational leadership rather than ethical leadership to predict employees’ ethical behavior is imperfect [26]. Compared with transformational leadership, ethical leadership can better reflect the ethical quality of leaders. Hence, this article attempts to explore the influence mechanism of ethical leadership on employees’ SRB, examining the mediating effect of perceived organizational virtuousness between them. For clarity, our research framework is summarized in Figure 1.

In this research, we adopt Roeck and Farooq’s [7] definition of employee’s SRB, and considered this construct consisting of two dimensions: pro-environmental behavior, including employees’ actions to perform work in an environmentally friendly way (e.g., recycling, rational use of resources, participating in environmental initiatives); philanthropic behavior, including employees’ actions that support overall community well-being (e.g., charitable donations).

In Figure 1, employees’ perceived ethical leadership, employees’ perceived organizational virtuousness, and employees’ SRB are individual-level variables. Ethical leadership is a group-level variable, the measurement of which is based on the upward integration of employees’ ethical leadership perception. In the field of organizational behavior, a common practice to measure group-level variables is, selecting some employees randomly from a group, investigating these employees’ perception about the above variables, under the condition that the internal consistency among individual perceptions in each group meet the requirement, the individual-level data is integrated into the group-level by means of the average. In our model, we propose that ethical leadership has a direct cross-level effect on employees’ SRB. We further propose that ethical leadership has an indirect cross-level effect on employees’ SRB with employees’ perceived organizational virtuousness (POV) playing a mediating role.

## 3. Hypothesis

### 3.1. The Direct Effect of Ethical Leadership

Ethical leadership can be defined as the demonstration of normatively appropriate conduct through personal actions and interpersonal relationships, and the promotion of such conduct to followers through two-way communication, reinforcement, and decision making [27]. To be perceived as ethical leaders, leaders should play two roles: a moral person and a moral manager [28]. We believe that both of two roles played by ethical leaders can enhance employees’ SRB.

Nowadays, environmental and charitable consciousness has gradually been incorporated into socially ethical norms in many countries [29]. As a moral person, ethical leaders hold a high ethical orientation, and will take the lead in abiding by the ethical norms of the society [28]. Furthermore, because of the ethical attribute of environmental protection and charitable donation, these behaviors have become the characteristic behavior of ethical leaders. For example, ethical leaders pay attention to the ethical consequences and long-term risks associated with decisions that go against the interests of various stakeholders [30]. Many scholars believe that philanthropy is an important pursuit of ethical leaders, because charitable acts by leaders demonstrate their altruism, their commitment to improving society, and their commitment to higher goals beyond their own or organizational interests [31]. According to the social learning theory, human behavior is mainly acquired through direct experiential learning and indirect learning by observing the behavior of role models [32]. Therefore, if business leaders behave environmentally friendly and pro-socially, employees can also adopt environmentally friendly behavior and charitable behavior by imitating leaders. Through leading by example, such as voluntarily sharing information regarding ecological issues, enhancing employee knowledge and understanding, valuing feedback, and encouraging people in solving environmental issues, it is reasonable to think that ethical leaders will influence their employees’ discretionary sense of attachment and responsibility to environmental concerns [12]. Moreover, ethical leaders strive to balance the various needs of stakeholders in a way that serves the interests of all, and therefore they often appear as CSR champions who demonstrate and promote SRB to their followers [7].

As a moral manager, ethical leaders urge employees to abide by ethical rules and to behave ethically through various management measures, such as communication, incentives, and cultural construction [28]. Recent studies showed that ethical leaders implement values-based management, communicate ethical standards to employees, and develop clear ethical policies and programs, which are associated with CSR activities in organizations [33]. By formulating the ethical norms in the organization and rewarding or punishing the members’ behavior accordingly, ethical leaders can make every employee clearly aware of the importance and necessity of ethical behavior. Ethical leadership attaches great importance to ethical management, which can establish a strong ethical atmosphere, stimulate employees’ moral identity, and enhance employees’ moral cognitive stage [34]. According to the theory of planned behavior, subjective norm is an important determinant of individual behavior intention [35]. Subjective norm means the extent to which individuals believe that they are under social pressure to perform the behavior. Therefore, if the organizational norm is to be environmentally friendly, employees will adopt environmentally friendly behavior by immersing themselves in such an atmosphere. Empirical research shows that subjective norms of environmental protection do significantly predict employee’s intentions to engage in pro-environmental behavior [36]. In short, through role modeling and ethical management, ethical leaders give employees impetus to the pursuit of social responsibility initiatives. Therefore, we can propose the following hypothesis:

**H1:**  *Ethical leadership has a positive effect on employees’ SRB.*

### 3.2. The Indirect Effect of Ethical Leadership

#### 3.2.1. Organizational Virtuousness

‘Virtuousness’ is a concept strongly related to but different from the concept of ‘virtue’ [37]. Virtues are human character traits—habituated patterns of thought, emotion, motivation or volition, and action that are consistently morally excellent and develop well-being [38]. Virtuousness refers to a constellation of virtues in the aggregate level [39]. Virtuousness is an ethos of virtuous character and a second-order construct, while virtue is a first-order construct [40]. Although virtue and virtuousness are sometimes considered relevant only for individuals, some have justified the rationale and applied the concept to organizations (e.g., [41,42]). Currently, it is widely accepted that both concepts can be used for analysis at both individual-level (i.e., personal virtuousness or virtue) and organizational-level (i.e., organizational virtuousness or virtue) [43].

Organizational virtuousness refers to a constellation of virtues in the aggregate, as organizations—similarly to individuals—display more than one virtue [44]. Optimism, forgiveness, compassion, trust and integrity have been found to be those virtues whose combination can capture the concept of organizational virtuousness from an employee point of view [45].When we speak of an organization being virtuous, we are not referring to the virtues of its members, but rather we are treating the organization as a unified organism with its own deliberative systems, structures, processes, and culture such that the organization itself has virtues [39]. Organizational virtuousness can be manifested in individual actions, collective actions, aspects of organizational culture and structure, and processes that encourage the enactment of virtuousness [44]. In this study, we conceptualize organizational virtuousness as an aggregate construct, and treat the aforementioned five virtues as dimensions of the multidimensional construct of organizational virtuousness, as well as focus on organizational virtuousness as perceived by employees (POV).

#### 3.2.2. Ethical Leadership and POV

Moore and Beadle [42] argued that the first precondition for a virtuous business organization is the presence of virtuous agents at the level of both the practice and the institution. Without agents who possess and exercise the virtues, the practice itself would no longer be fostered internally through the pursuit of excellence, and at the institutional level the corruption of the institution and the consequent distortion of the practice would seem to be inevitable. This is particularly true for agents with decision-making authority in the institution [42]. Moore believed that the cultivation of organizational virtuousness can’t be separated from a favorable internal governance system, one of the key characteristics of which is that the organization has virtuous agents [46].

Managers play important roles in fostering organizational virtuousness. Ethical leaders can be called virtuous agents. They are honest and principled leaders who seek to do the right thing and conduct their lives in a moral way. They tend to make fair and balanced decisions and work from the means perspective rather than the ends perspective [47]. In a manner consistent with virtue ethics, ethical leadership behavior is conceptualized as acting in a manner that communicates the importance of considering the means by which outcomes are achieved [27]. They set, communicate and implement clear ethical standards among their subordinates. Managers who engage in ethical leadership behavior act as virtuous agents in promoting an ethical climate [48]. Thus, ethical leadership will contribute to not only the cultivation of organizational virtuousness, but also the formation of employees’ POV. Therefore, we expect that:

**H2:**  *Ethical leadership positively predicts employees’ POV.*

#### 3.2.3. POV and Employees’ SRB

Many scholars believe that perceived organizational virtuousness can increase employees’ organizational identification, lead them to experience positive emotions, and guide them to engage in organizational citizenship behavior. For example, Rego, Ribeiro and Cunha found that employees’ POV predict some OCB both directly and through the mediating role of their affective well-being [49]. POV may also lead employees to develop relational psychological contracts with the organization, thus reacting with behaviors that go beyond their in-role duties, which benefit the organization [49]. The feeling of working in a virtuous organization may encourage employees to work not only for financial rewards or career advancement, but also for the personal gratification of “doing a good job”. Adopting OCB is a way to perform such a “good job” [49].

Perceiving organizational virtuousness helps unlock the human predisposition to behave in ways that benefit others and can make employees develop pro-social motives toward their organization [50]. According to Cameron [50], organizational virtuousness can foster pro-social behavior through the activation of individuals’ pro-social motives. Observing organizational virtuousness can activate individuals’ internal definitions of goodness and can make them desire to behave in a similar way and benefit others [44,50]. In other words, perceiving organizational virtuousness can intrinsically motivate individuals by activating their pro-social motives [51]. The findings of the experimental study of Tsachouridi and Nikandrou indicate that organizational identification mediates the positive relationship between POV and organizational spontaneity [52]. Cameron et al. argued that virtuousness has contagious effect [45]. When individuals perceive virtuousness, an irresistible impulse is generated to engage in virtuous behavior. All the above arguments indicate that perceived organizational virtuousness is positively correlated with employees’ pro-social behavior. Because employees’ SRB also has the nature of pro-social behavior, the following is proposed:

**H3:**  *POV positively predicts employees’ SRB.*

#### 3.2.4. The Mediating Role of POV

The POV belongs to the domain of psychological climate in essence. Psychological climate helps people explain events, predict possible outcomes and measure the rationality of their subsequent actions. Scholars generally believe that psychological climate is an individual’s direct or indirect perception of a particular environment. It can affect the motivation, attitude, belief and values of employees in an organization. It is appropriate to use psychological climate to predict employees’ SRB, because it is people’s subjective perception and evaluation of the environment (rather than the objective environment itself) that determines people’s follow-up behavior. For example, James and James pointed out that individual behavior is not directly affected by the objective environmental characteristics, but by the individual’s interpretation of the environment [53]. Atmosphere perception mediates the relationship between the objective environment of an organization and individual behavior.

Ethical leadership helps establish a virtuous organizational environment and cultivate a virtuous organizational personality. In other words, ethical leaders’ behavior sends a signal to employees everywhere that they are expected and encouraged to do ethical things. Next, employees may perceive a virtuous organizational environment and form an ethical psychological climate. When an organization’s environment is perceived as ethical or virtuous, these perceptions will affect the ethical behavior of its members [47]. Also, according to the contagious effect of virtuousness, employees who perceive organizational virtuousness will spontaneously exhibit ethical behavior, including extra-role behavior [45]. In short, if ethical leadership is distributed at all levels of the organization, then the organization will be permeated with a strong ethical atmosphere and a variety of ethical behavior. This can help people form the sense of organizational virtuousness and thus induce more pro-social behavior. Lu and Lin showed the mediating effect of ethical climate on the relationship between ethical leadership and employee ethical behavior [54]. Choi et al. also found that employees’ perception of an ethical work climate will act positively as a mediator in the relationship between ethical leadership and followers’ attitudes toward CSR [55]. Therefore, the influence of ethical leadership (organizational context variables) on employees’ SRB (individual behavior variables) is transmitted through employees’ perception of organizational virtuousness (psychological climate). Based on the above analysis, this following is proposed:

**H4:**  *POV mediates the influence of ethical leadership on employees’SRB.*

## 4. Methods

### 4.1. Samples

In this research, we focus on cross-level analysis between group and individual levels. To obtain the required data from a large number of firms, we asked the on-the-job undergraduate students of a distance-learning university in Shanghai to help us contact their department managers. These students are widely distributed in different firms and industries located in various regions and provinces in China including Sichuan, Shandong, Shanghai, Zhejiang, Jiangsu, Guangdong, Ningxia and Chongqing. Through these distance-learning students we were able to reach 109 department managers in different companies. Under the permission of each of those managers, we randomly invited some employees from his/her department to answer a questionnaire of perceived organizational virtuousness and perceived ethical leadership. The questionnaires with a cover letter indicating the purpose of the investigation were sent to the selected employees through various methods such as emails and physical mails. Finally, we received 936 valid questionnaires. The number of participants in each department ranges from 3 to 11. After that, we asked the department managers to answer the questionnaire of SRB for those selected employees one by one. Hence in this study, the data of independent variables and mediating variables are from employees, and the data of dependent variables are from employees’ managers. In this way, the problem of common method biases in measuring can be avoided.

The survey work lasted about two years from 2014 to 2016. The following industries are involved: food and beverage (22 firms), machinery manufacturing (35 firms), bio-pharmaceutical (13 firms), textile and paper (12firms), petrochemical (threefirms), power generation (fourfirms), IT technology (20 firms). The data used for analysis came from 109 managers and 936 employees. Of these 936 employees, 41.98% were men. In terms of age, 33.55% were under 25 years old, 37.39% were 26–30 years old, 16.88% were 31–35 years old, 6.62% were 36–40 years old, and 5.56% were over 41 years old. In terms of education, junior high school and below accounted for 0.75%, senior high school for 13.03%, college for 41.98%, undergraduate for 38.67%, and postgraduate and above for 5.56%. In terms of tenure, 17.31% are under one year, 27.67% are within one to two years, 33.55% are within two to five years, 15.17% are within five to ten years, and 6.31% are over ten years. In terms of positions, 70.62% of respondents are front-line employees and 29.38% of respondents are supervisors.

### 4.2. Measurements

Respondents completed the questionnaire in Chinese. The scales were originally developed in English; therefore, we translated all the items using the standard translation–back-translation procedure based on the International Test Commission guidelines. All items were administered on a five-point Likert scale (1 = strongly disagree, 5 = strongly agree).

#### 4.2.1. Ethical Leadership

Ethical leadership is a group-level variable and its measured value is obtained from the upward integration of ethical leadership perception at the individual level. Therefore, here we mainly discuss the formation of perceived ethical leadership scale. Brown et al.’s [27] the ethical leadership scale (ELS), a ten-item instrument, was used to measure perceived ethical leadership (see Box 1).

Box 1 **The perceived ethical leadership scale.**

Our manager listens to what employees have to say.

Our manager disciplines employees who violate ethical standards.

Our manager conducts his/her personal life in an ethical manner.

Our manager has the best interest of employees in mind.

Our manager makes fair and balanced decisions.

Our manager can be trusted.

Our manager discusses business ethics or values with employees.

Our manager sets an example of how to do things the right way in terms of ethics.

Our manger defines success not just by results but also the way that they are obtained.

When making decisions, our manager asks “what is the right thing to do?”

#### 4.2.2. Perceived Organizational Virtuousness

The 15-item scale proposed by Cameron et al. [45] was used to measure our mediating variable, perceived organizational virtuousness. In this scale, five cardinal virtues (optimism, trust, compassion, forgiveness, and integrity) are designed to represent the five dimensions of virtuousness (see Table 1).

#### 4.2.3. Employees’ SRB

In this study, employees’ SRB includes two aspects, employees’ pro-environmental behavior and employees’ philanthropic behavior. Roeck and Farooq [7] has proposed two scales to measure employees’ green behavior and societal behavior. Based on these two scales, we have developed an instrument to measure employees’ SRB (see Box 2). This instrument includes six items, three of which were used to measure pro-environmental behavior and the other three were used to measure philanthropic behavior.

Box 2 **Employees’ SRB scale.**

I adequately complete assigned duties in environmentally friendly ways.

I perform job tasks that are expected from me in environmentally friendly ways.

I take initiatives to act in environmentally friendly ways at work.

I give adequate contributions to charities and donations.

I am involved in social and volunteer work that benefits my community.

I engage myself in social and humanitarian causes and associations.

In this paper, tenure, age and education are taken as controlled variables. The literature indicated that these factors are significantly correlated with employees’ SRB (e.g., [7]). The specific evaluations of those controlled variables are as follows. Tenure is divided into five grades, 1 point for “less than one year”, 2 points for “1–2 years”, 3 points for “2–5 years”, 4 points for “5–10 years”, and 5 points for “more than 10 years”. Age is divided into five grades, one for “25 years old and below”, two for “26–30 years old”, three for “31–35 years old”, four for “36–40 years old”, and five for “41 years old and above”. Education is also divided into five grades, “junior high school and below” 1 point, “senior high school” 2 points, “junior college” 3 points, “undergraduate” 4 points, and “graduate students and above” 5 points.

## 5. Results

### 5.1. Reliability and Validity Analysis

Although the measures used in the questionnaire were already applied in the literature, before testing the proposed hypothesis, it is important to ensure that the constructs were empirically validated [56].

Composite reliability (CR) and corrected item–total-correlation (CITC) were estimated to perform the reliability analysis. As shown in Table 2, the CR value of all latent variables are higher than 0.70 and the CITC values of all latent variables exceeded the recommended value of 0.30. Thus, it is concluded that the scales were internally reliable and there are no construct reliability concerns of the measurement model. Moreover, all item loadings are higher than the recommended value 0.7, suggesting acceptable indicators reliability.

Our results of reliability test show that the scale for perceived ethical leadership has satisfactory reliability as Cronbach α is 0.916. The scale for employees’ SRB also has satisfactory reliability as Cronbach α is 0.856. Meanwhile, each dimension of POV as well as the construct of POV as a whole has satisfactory reliability as Cronbach α surpassed 0.70 (optimism = 0.811, trust = 0.797, Compassion = 0.805, integrity = 0.868, forgiveness = 0.816, and POV = 0.927).

Next, a confirmatory factor analysis (CFA) using LISREL and maximum likelihood estimation was used to test the discriminatory validity of the three research scales. As mentioned above, the ethical leadership scale includes one dimension, the employee’s SRB scale contains one dimension and the POV scale contains five dimensions. There are seven dimensions in total. Therefore, we construct a first-order seven-factor model and conduct a fitting test with 936 samples. The fitting index of the model is as follows: Χ2/df = 4.052, RMSEA = 0.057, SRMR = 0.039, NNFI = 0.980, CFI = 0.980 and GFI = 0.890, indicating a good alignment for the measurement model.

Furthermore, factor loadings of each construct higher than 0.70, CR values are higher than 0.80 (see Table 2). As shown in Table 3, AVE (average variance extracted) values for each construct are higher than 0.50, MSV (maximum shared variance) values are lower than AVE and the square root of AVE is higher than inter-construct correlations. These results from CFA show that the three variables in our study satisfy the required condition of convergent validity and discriminatory validity.

However, although POV contains five dimensions, we do not discuss these five dimensions separately in the following analysis. Rather, we regard the average score of these five dimensions as the POV score.

### 5.2. Aggregate Analysis

Ethical leadership is a group-level abstract variable and its measured value is obtained from the upward integration of ethical leadership perception at the individual level. Therefore, we need to aggregate the individual-level data to group level after justifying within-group agreement (r_wg_) and intra class correlation coefficient ICC (1) and ICC (2). In general, only when the median or mean of r_wg_ is greater than 0.70 [57], the ICC (1) is less than 0.5 and F test is significant, and the ICC (2) is over 0.7 [58], can we aggregate the individual-level data to an upper level.

Taking the responses from the same company as a group, we divided the sample data into 109 groups. We first computed the r_wg_ statistic for perceived ethical leadership for each group. The result showed that the r_wg_ of each group was greater than 0.70, with the minimum value of 0.732, the maximum value of 0.986, and the mean of 0.932. We then conducted a one-way ANOVA and computed the ICC (1) and ICC (2) statistics for perceived ethical leadership based on these 109 groups. The result showed that ICC (1) was 0.175 and ICC (2) was 0.646. Thus, the results of aggregate analyses showed that it was acceptable to aggregate the individual-level data for perceived ethical leadership to a collective level.

### 5.3. Common Method Bias Test and Descriptive Statistics

Before regression analysis, we applied the Harman’s one-factor test to examine common method bias. An exploratory factor analysis (EFA) was conducted involving all the observed variables considered. The results of the EFA revealed seven unique factors with eigenvalues above 1.0 and a cumulative variance equal to 63.17%. The first extracted factor explained the 41.91% of the variance, which was not the majority of the cumulative variance as it is below 0.50. Hence, common method bias is not serious in this study.

In this research, the variables involve individual level (Level-1) variables and group level (Level-2) variables. Level-1 variables include controlled variables (age, education and tenure), POV and employees’ SRB. The Level-2 variable is the ethical leadership. The descriptive statistical results of these are shown in Table 4.

### 5.4. Hierarchical Linear Model Analysis

Because data were nested (i.e., employees were nested within organizational units), we employed hierarchical linear modeling (HLM) to test the multilevel hypotheses. We employed the full maximum likelihood to estimate the parameters. Level-1 variables were group-mean centered, and Level-2 variables were grand-mean centered. According to Zhang et al. [59], the mediation of POV on the relationship between group ethical leadership and employees’ SRB was identified as the cross-level mediation-lower mediator (2-1-1 model). Our multilevel mediation analysis procedure followed Zhang et al.’s [59] recommendations. Moreover, the mediation analyses were conducted with the recommendations of Baron and Kenny [60], following the following four conditions: (a) independent variable must be related to dependent variable; (b) independent variable must be related to the mediator; (c) mediator must be related to the dependent variable; and (d) when independent variable and the mediator are included, the direct relationship between independent variable and the dependent variable should become less significant (partial mediation) or non-significant (full mediation).

Step 1: Null model

Since this study hypothesizes that employees’ SRB at individual level can be predicted by variables at individual level and group level, it must be shown that there are variations in the employees’ SRB at individual level and group level. Therefore, the first step is to divide the variance of employees’ SRB into intra-group variance and inter-group variance using null model without predictors. The model is as follows:

Level-1 Model
  SRB_ij_ = β_0j_ + r_ij_
Level-2 Model
  β_0j_ = γ_00_ + U_0j_

The analysis results from Step 1 are inter-group variance (τ_00_) = 0.040, χ^2^(108) = 202.685, and *p* < 0.001, indicating that the inter-group variance was significant. In addition, intra-group variance (σ2) = 0.390 and ICC (1) for SRB= τ_00_/(σ^2^+τ_00_) =0.093, indicating that 9.3% of the variance of SRB is the inter-group variance, while 90.7% is the intra-group variance. Since SRB has significant inter-group variance, hypothesis testing can be carried out next.

Step 2: Testing the direct effect of ethical leadership on SRB

In order to test the direct effect of ethical leadership on SRB, we take SRB as outcome variable, add controlled variables to Level-1, add ethical leadership to Level-2, and estimate the following models:

Level-1 Model
  SRB_ij_ = β_0j_ + β_1j_*(Age_ij_) + β_2j_*(Education_ij_) + β_3j_*(Tenure_ij_) + r_ij_
Level-2 Model
  β_0j_ = γ_00_ + γ_01_*(Ethical leadership_j_) + U_0j_
  β_1j_ = γ_10_ + U_1j_
  β_2j_ = γ_20_ + U_2j_
  β_3j_ = γ_30_ + U_3j_

In above models, γ_01_ represents an estimate of the relationship between ethical leadership and SRB. We can use *t*-test for γ_01_ to verify the direct effect of independent variables (ethical leadership) on dependent variables (SRB).In above models, γ_10_ represents an estimate of the relationship between age and SRB, γ_20_ represents an estimate of the relationship between education and SRB, and γ_30_ represents an estimate of the relationship between tenure and SRB. We use t-test for γ_10_, γ_20_ and γ_30_ to verify the effect of control variables on outcome variable. The analysis results of Step 2 are: γ_01_ = 0.462, t = 6.874, and *p* < 0.001, indicating that ethical leadership has a significant positive impact on SRB. Therefore, Hypothesis 1 is supported. Moreover, γ_10_ = 0.062, t = 2.255, and *p* < 0.050, indicating that age has a significant positive impact on SRB. γ_20_ = 0.138, t = 5.355, and *p* < 0.001, indicating that educational has a significant positive impact on SRB. Finally, γ_30_ = 0.058, t = 2.499, and *p* < 0.05, indicating that tenure has a significant positive impact on SRB.

Step 3: Testing the direct effect of ethical leadership on POV

In order to test the influence of ethical leadership on POV, we take POV as the outcome variable, add controlled variables to Level-1, add ethical leadership to Level-2, and estimate the following models:

Level-1 Model
  POV_ij_ = β_0j_ + β_1j_*(Age_ij_) + β_2j_*(Education_ij_) + β_3j_*(Tenure_ij_) + r_ij_
Level-2 Model
  β_0j_ = γ_00_ + γ_01_*(Ethical leadership_j_) + U_0j_
  β_1j_ = γ_10_ + U_1j_
  β_2j_ = γ_20_ + U_2j_
  β_3j_ = γ_30_ + U_3j_

In above models, γ_01_ represents an estimate of the relationship between ethical leadership and POV. We use *t*-test for γ_01_ to verify the effect of independent variables (ethical leadership) on mediating variables (POV). The analysis results of Step 3 are: γ_01_= 0.605, standard error = 0.067, t=9.051, and *p* < 0.001, indicating that ethical leadership positively affects POV, and the effect is significant. Therefore, Hypothesis 2 is supported.

Step 4: Controlling ethical leadership and testing the effect of POV on SRB

We take SRB as the outcome variable, add controlled variables and group-mean-centered POV to Level-1, add ethical leadership and group mean of POV to Level-2, and estimate the following models:

Level-1 Model
  SRB_ij_ = β_0j_ + β_1j_*(Age_ij_) + β_2j_*(Education_ij_) + β_3j_*(Tenure_ij_) +β_4j_*(POV_ij_ − POV_j_) + r_ij_
Level-2 Model
  β_0j_ = γ_00_ + γ_01_*(Ethical leadership _j_) + γ_02_*(POV_j_) + U_0j_
  β_1j_ = γ_10_ + U_1j_
  β_2j_ = γ_20_ + U_2j_
  β_3j_ = γ_30_ + U_3j_
  β_4j_ = γ_40_ + U_4j_

In above models, POV_j_ represents group mean of POV, and γ_01_ represents an estimate of the relationship between ethical leadership and SRB. We conduct *t*-test for γ_01_ to verify the effect of independent variable (ethical leadership) on dependent variable (SRB) after adding mediating variable (POV). In addition, γ_02_ represents an estimate of the relationship between POV and SRB. We conduct t test for γ_02_ to verify the effect of mediating variables (POV) on dependent variables (SRB).The analysis results of Step 4 are: γ_02_ = 0.379, standard error = 0.094, t = 4.020, and *p* < 0.001, indicating that POV positively affects employees’ SRB, and the effect is significant. Hence, Hypothesis 3 is supported. In addition, we have γ_01_ = 0.227, t = 2.543, and *p* < 0.050, indicating that the effect of ethical leadership on employee’s SRB decreases when POV is added (because in the second step of direct effect test, γ_01_ = 0.462, t = 6.874, and *p* < 0.001).

In conclusion, the results from Step 1 show that ethical leadership has a significant direct effect on employees’ SRB. The results of Step 4 show that the effect of ethical leadership on employees’ SRB decreases after adding POV. Therefore, POV plays a mediating role between ethical leadership and employees’ SRB. Therefore, Hypothesis 4 is supported. Next, we use Sobel test to examine the significance of mediation effect. The results show the value of statistic *Z* as 3.664, indicating that the mediating effect of POV is significant at the level of 0.01.

## 6. Discussion

### 6.1. Theoretical Implications

First, for the long-term survival of man and nature, we must act responsibly and consider environment in our day-to-day organizational life [61]. Most existing studies have linked ethical leadership to the followers’ ethical conduct (such as OCB) and unethical conduct (such as counterproductive behavior) [62]. However, less research attention has been paid to the effect of ethical leadership on employees’ SRB which can contribute to organizational sustainable development and social change.Our study emphasizes those pro-environmental and philanthropic activities of employees in their daily organizational life. Although the effect of these activities on organizational sustainable development and social change seems to be negligible when viewed independently, it will be considerable when a large number of employees do similar things [8].

Second, in existing studies, ethical leadership is usually perceived as constructs at individual level, emphasizing the impact of employees’ perception of ethical leadership on their attitudes and behaviors. However, more and more scholars proposed that leadership was more than individual perception in the sense that it could be the group process that referred to the collective belief about the leaders’ traits and behaviors, and recommended to examine the impacts of leadership at multilevel levels [62]. With the development of multilevel methodologies, researchers have shifted their attention to the exploring and testing of multilevel construct. In this research, we answer this call and treat ethical leadership as a group-level concept in our models.

Third, a large number of studies have shown that employees’ perception of organizational virtuousness can affect employees’ attitudes and behaviors. For example, Chun showed that organizational virtuousness perception is positively correlated with internal employees’ overall satisfaction with the company and external customers’ overall satisfaction with the company [63]. Chun showed that employees’ perceived virtuousness of the merged company is positively correlated with employees’ general satisfaction, emotional attachment, job security and loyalty [64]. Despite its importance, there is a lack of research on the determinants of organizational virtuousness perception. This study examined the impact of ethical leadership on organizational virtuousness perception, thus filling the research gap.

Finally, we contribute to the research on the relationship between leadership style and employees’ socially responsible behaviors. As a matter of fact, the role of perceived organizational virtuousness as an explanatory mechanism of the ethical leadership-outcome relationship has been rarely examined in the literature.

### 6.2. Practical Implications

In order to achieve the sustainable development of society, we should pay attention not only to enterprises’ socially responsible behavior, but also to nurturing and shaping employees’ daily socially responsible behavior. In this research, we focus on employees’ activities of environmental protection and charitable donation. We obtain the following managerial implications.

First of all, leaders, as "important others" of employees in the workplace, can play a key role in the process of shaping employees’ behavior. Especially, our findings confirm that ethical leadership style helps employees demonstrate behaviors of environmental protection and charitable donation. Managers who display ethical leadership qualities such as integrity and fairness, who reward and support employees who behave ethically, and who emphasize ethical standards and serve as ethical behavior role models, are better equipped to create an ethical climate in which doing the right thing is of value [54]. Therefore, in management practice, enterprises should attach importance to the selection and training of leaders, enhance their own ethical awareness and moral level, and guide them to adopt ethical leadership. This can greatly facilitate employees’ SRB.

Secondly, in order to encourage employees’ environmental protection and charitable behavior, enterprises can design appropriate environmental protection and donation policies as guidelines. However, formal policies cannot be comprehensive and employees’ spontaneous environmental and charitable behavior is greatly needed. Hence, when hiring, enterprises can aim to hire more employees with pro-social and environmental values.

Last but not least, our findings confirm that perceived organizational virtuousness has a positive impact on employees’ SRB of environmental protection and charitable donation. Therefore, in order to promote an employee’s motivation in taking SRB, it is critical for managers to cultivate organizational virtuousness perception in employees. For this purpose, managers should maintain an ethical work climate in which employees are concerned about the interests of stakeholders and the society [55]. For one thing, mangers can establish ethical standards, communicating the importance of ethics and rewarding and supporting employees who behave ethically or serve as role models of ethical behavior. For another thing, what matters more is that managers walk the talk by displaying ethical and pro-social behaviors [29]. In addition, mangers can promote ethical perception among employees through public relations activities as well as internal company activities [26].

### 6.3. Future Research

First, in this study, we have explored whether and how ethical leadership influences employees’ SRB. However, we do not know whether the impact of ethical leadership on employees’ SRB is moderated by situational or individual factors. Avey et al. have examined the moderating effect of self-esteem between ethical leadership and employees’ organizational citizenship behavior [65]. Their results show that the effect of ethical leadership on organizational citizenship behavior decreases with the improvement of individual self-esteem. Therefore, future research can explore the interactive effect between ethical leadership and individual factors on employees’ SRB. After all, employee behavior is the result of the interaction between personal factors and environmental factors. To motivate employees to care more for the environment and serve for society, leaders’ behavior style and employees’ internal qualities need to work together.

Second, ethical leadership is helpful not only for employees to form organizational virtuousness perception, but also for firms to form virtuous culture. Payne et al. defined the concept of organizational virtue orientation (OVO) which is described as an organization’s integrated set of values and beliefs supporting ethical character traits and virtuous behaviors [66]. The difference between organizational virtue orientation and organizational virtue lies in that the former relates to the assumptions and values that support o ethical behavior, while the latter relates to the actual activities of ethical behavior. This study has examined the mediating effect of perceived organizational virtuousness between ethical leadership and employees’ SRB. Thus, future research can explore the mediating effect of organizational virtue orientation between the two.

Third, due to the proximity between department leaders and employees, managers have a very direct impact on employees. So, our empirical study focused on the influence of the leadership style of departmental managers on employees’ SRB. Yet, because leaders at the organizational level, such as CEO, have more power in establishing organizational culture and formulating corporate policies, hence, it is worthwhile for future researchers to explore the relationship between CEO ethical leadership and employees’ SRB.

Last but not least, Searle and Barbuto have argued that servant leadership which is characterized by organizational stewardship and altruistic calling is related to the development of organizational virtuousness [67]. Therefore, future research can explore the effect of servant leadership on perceived organizational virtuousness and employees’ SRB.

## 7. Conclusions

Solving the various environmental and societal problems needs the development of science and technology as well as behavior changes of people. In this study, we focus on if and how ethical leadership can affect employee’s socially responsible behaviors of environmental protection and charitable donation. Specifically, we explore the cross-level direct effect of ethical leadership on employees’ socially responsible behaviors and the cross-level mediating effect of perceived organizational virtuousness between them. Our empirical results show that perceived organizational virtuousness partly mediates the influence of ethical leadership on employees’ socially responsible behaviors of environmental protection and charitable donation. Stated differently, ethical leadership enables employees to form organizational virtuousness perception. Hence, more socially responsible behaviors of environmental protection and charitable donations are displayed by employees because of the contagious effect of virtuousness.

## Figures and Tables

**Table 1:** The perceived organizational virtuousness scale.

Optimism
We are optimistic that we will succeed, even when faced with major challenges.
In this organization, we are dedicated to doing good in addition to doing well.
A sense of profound purpose is associated with what we do here.
Trust
Employees trust one another in this organization.
People are treated with courtesy, consideration, and respect in this organization.
People trust the leadership of this organization.
Compassion
Acts of compassion are common here.
This organization is characterized by many acts of concern and caring for other people.
Many stories of compassion and concern circulate among organization members.
Integrity
This organization demonstrates the highest levels of integrity.
This organization would be described as virtuous and honorable.
Honesty and trustworthiness are hallmarks of this organization.
Forgiveness
We try to learn from our mistakes here, consequently, missteps are quickly forgiven.
This is a forgiving, compassionate organization in which to work.
We have very high standards of performance, yet we forgive mistakes when they are acknowledged and corrected.

**Table 2:** Measurement model results.

Latent Variable		Indicators	Standardized Factor Loadings	CR	CITC
Perceived Ethical leadership		ELS1	0.73	0.93	0.62–0.78
		ELS2	0.76		
		ELS3	0.74		
		ELS4	0.80		
		ELS5	0.83		
		ELS6	0.81		
		ELS7	0.74		
		ELS8	0.78		
		ELS9	0.73		
		ELS10	0.75		
Perceived organizational virtuousness (POV)	Optimism	OPT1	0.81	0.81	0.61–0.68
		OPT2	0.79		
		OPT3	0.71		
	Trust	TRU1	0.72	0.80	0.63–0.67
		TRU2	0.80		
		TRU3	0.75		
	Compassion	COM1	0.69	0.80	0.62–0.66
		COM2	0.82		
		COM3	0.77		
	Integrity	INT1	0.83	0.85	0.73–0.78
		INT2	0.84		
		INT3	0.81		
	Forgiveness	FOR1	0.76	0.81	0.65–0.69
		FOR2	0.83		
		FOR3	0.72		
Socially responsible behavior (SRB)		SRB1	0.82	0.89	0.63–0.75
		SRB2	0.82		
		SRB3	0.79		
		SRB4	0.71		
		SRB5	0.76		
		SRB6	0.71		

CR: composite reliability; CITC: corrected item-total-correlation.

**Table 3:** Validating the measurement of CFA model.

	AVE	MSV	ELS	OPT	TRU	COM	INT	FOR	SRB
ELS	0.59	0.394	0.768						
OPT	0.59	0.365	0.509	0.768					
TRU	0.57	0.484	0.607	0.598	0.755				
COM	0.58	0.461	0.543	0.425	0.641	0.762			
INT	0.68	0.484	0.628	0.489	0.696	0.679	0.825		
FOR	0.59	0.404	0.571	0.543	0.584	0.571	0.636	0.768	
SRB	0.59	0.365	0.573	0.604	0.508	0.466	0.486	0.483	0.768

CFA: confirmatory factor analysis; AVE: average variance extracted; MSV: maximum shared variance; ELS: ethical leadership; OPT: optimism; TRU: trust; COM: compassion; INT: integrity; FOR: forgiveness; SRB: socially responsible behavior.

**Table 4:** The descriptive statistics of the variables.

Variables	Sample Size	Minimum	Maximum	Mean	Standard Deviation
Level-1					
Age	936	1.000	5.000	2.130	1.120
Education	936	1.000	5.000	3.350	0.800
Tenure	936	1.000	5.000	2.650	1.120
POV	936	1.000	5.000	3.940	0.660
Employees’ SRB	936	1.000	5.000	3.780	0.650
Level-2					
Ethical leadership	109	2.330	4.640	3.760	0.390
